# Supplementary material for: In Vitro versus in Mice: Efficacy and Safety of Decoquinate and Quinoline-O-Carbamate Derivatives against Experimental Infection with Neospora caninum Tachyzoites
Source: Pathogens. 2023 Mar 13;12(3):447. doi: 10.3390/pathogens12030447 (PMC10055983; doi:10.3390/pathogens12030447)
Supplement: Supplementary file 1 [file pathogens-12-00447-s001.zip › pathogens-2266997-supplementary.pdf]

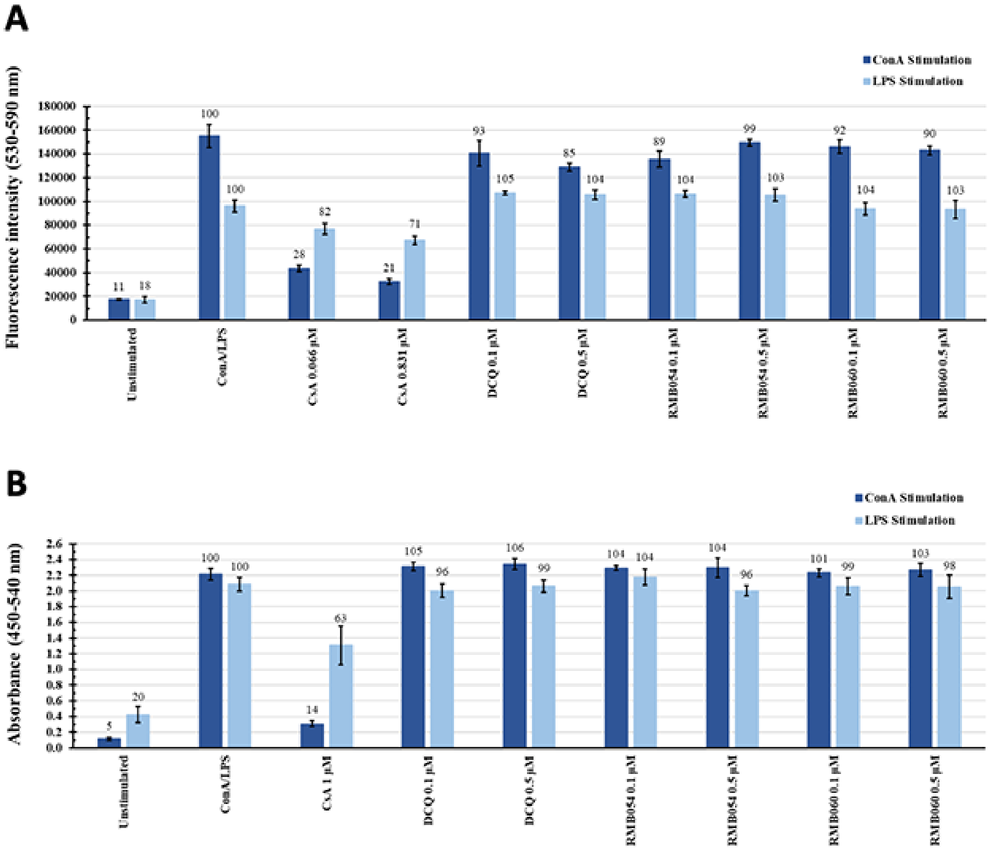

Figure S1: Viability assessment (A) and proliferative capacity (B) of ConA- and LPSinduced T- and B-lymphocytes treated with DCQ, RMB054 and RMB060 (0.1 and 0.5 µM) measured by Alamar Blue assay (A) and BrdU incorporation ELISA (B). Data in (A) are presented as means of the fluorescence intensity of four replicates ± standard deviation, in (B) the data are presented as means of the absorbance of three replicates ± standard deviation. The percentage of viability/proliferation in relation to the non-drug treated control is indicated in the graph for each sample. Dark blue bars represent viability/proliferation of ConA activated T-cells and light blue bars of LPS activated Bcells. A full viability/proliferation (100%) was attributed to spleen cells only stimulated with ConA or LPS and served as absolute control for non-effect of the drug treatment. Cyclosporin A (CsA) was used for its reported immunosuppressive properties and served as control for presence of inhibitory effect on viability of immune cells.

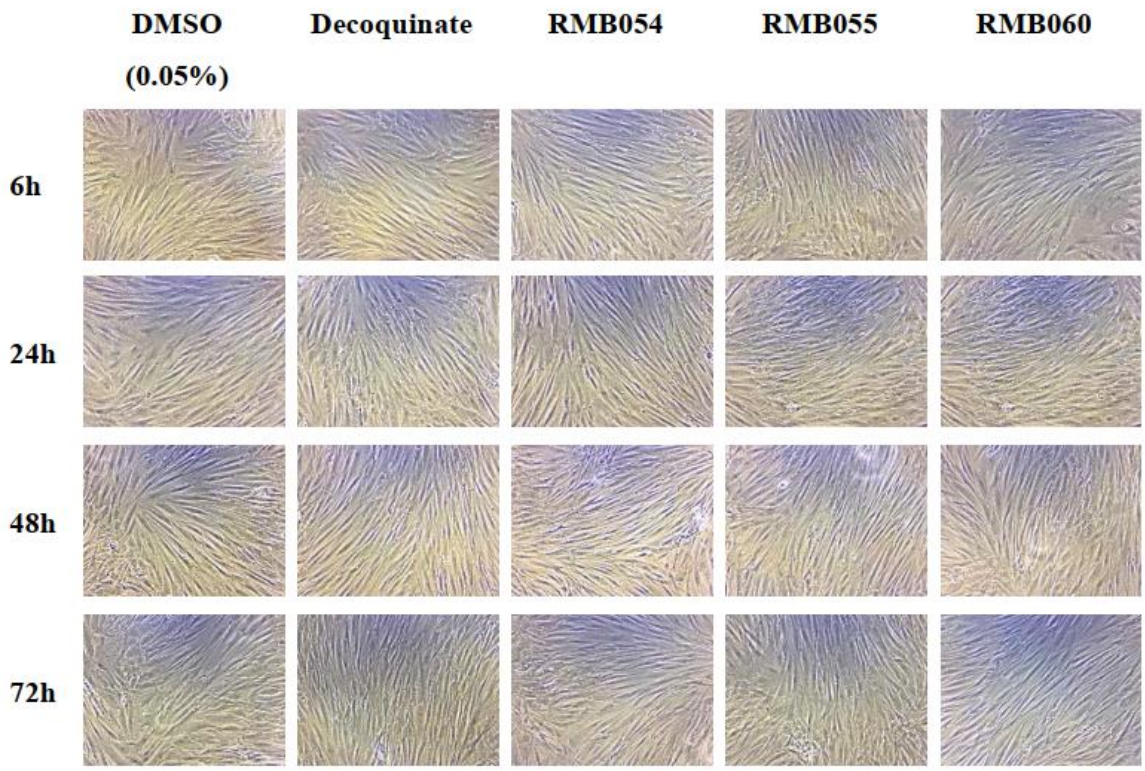

Figure S2: HFF cultures exposed to 0.5 μM DCQ, RMB054, RMB055 and RMB060 for 6, 24, 48 and 72 hours in comparison to DMSO (0.05%) as a negative control.

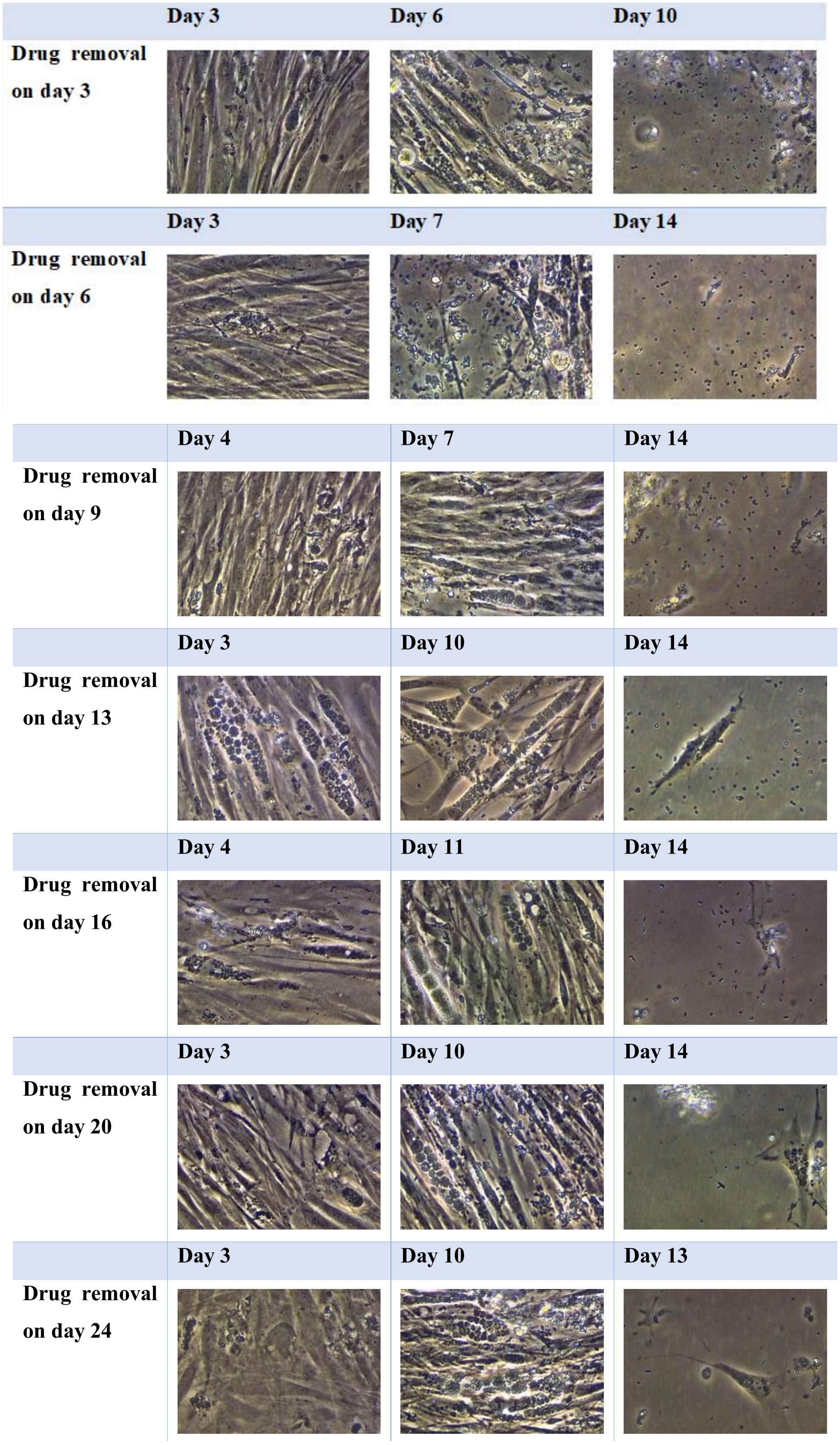

Figure S3: Long term treatment of *N. caninum* Spain-7 with 0.5 µM DCQ for different consecutive time spans On the days indicated on the left, drug pressure was removed to assess parasiticdial versus parasitostatic activity. Images were taken on the days of culture after drug removal indicated on the top of the micrographs

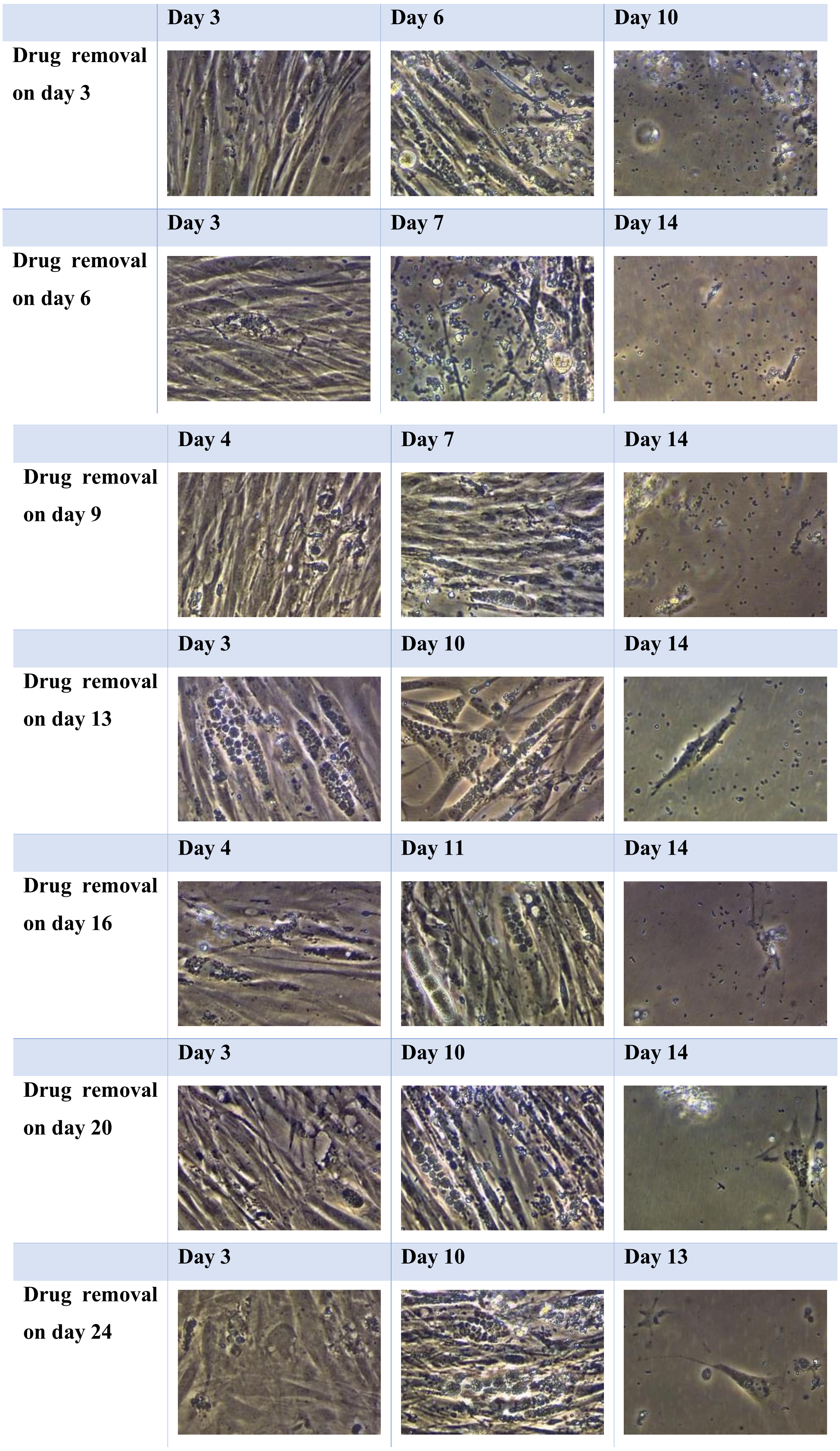

Figure S4: Long term treatment of *N. caninum* Spain-7 with 0.5 μM RMB054 for different consecutive time spans. On the days indicated on the left, drug pressure was removed to assess parasiticdial versus parasitostatic activity. Images were taken on the days of culture after drug removal indicated on the top of the micrographs

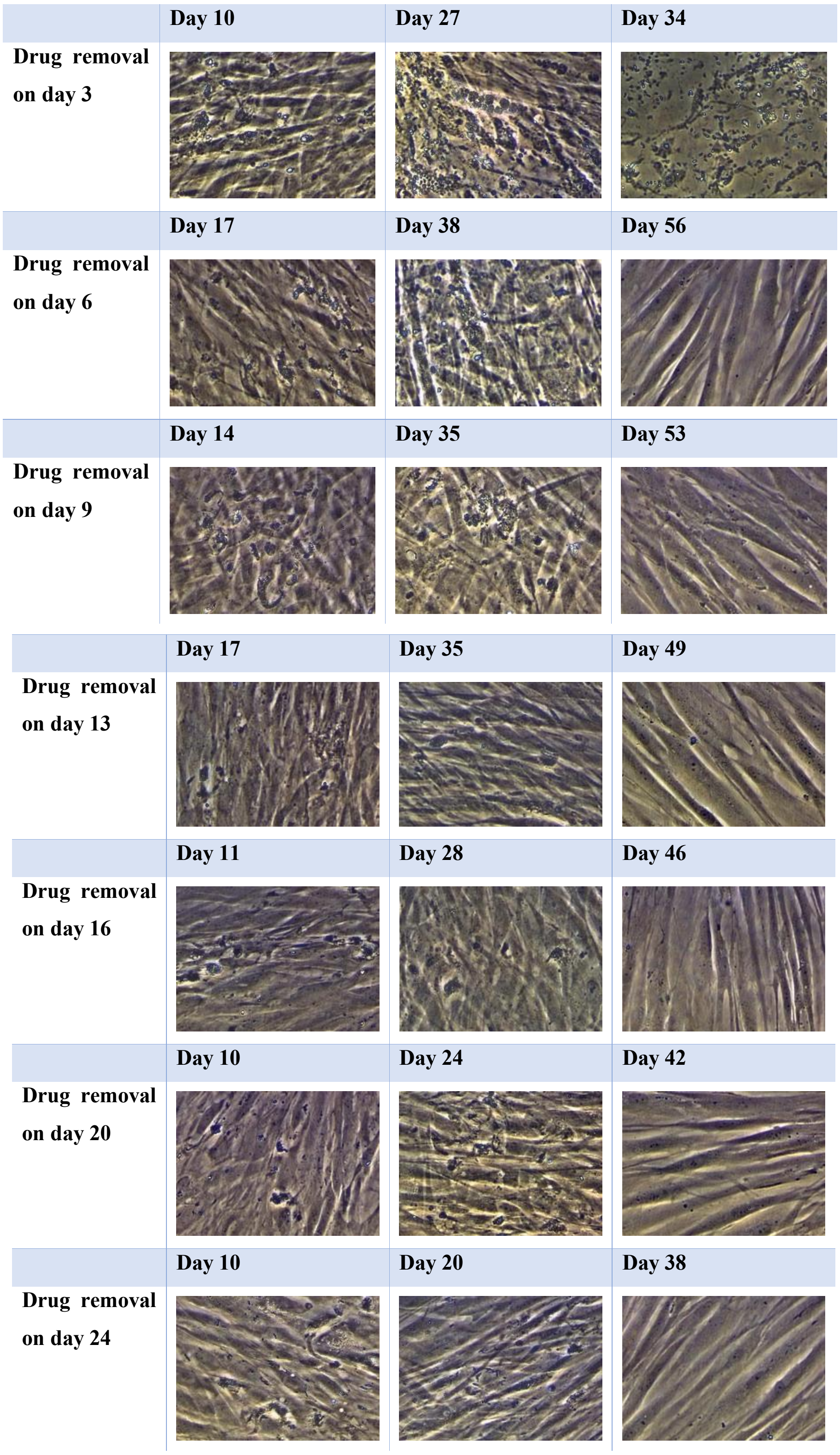

Figure S5: Long term treatment of *N. caninum* Spain-7 with 0.5 μM RMB060 for different consecutive time spans. On the days indicated on the left, drug pressure was removed to assess parasiticdial versus parasitostatic activity. Images were taken on the days of culture after drug removal indicated on the top of the micrographs

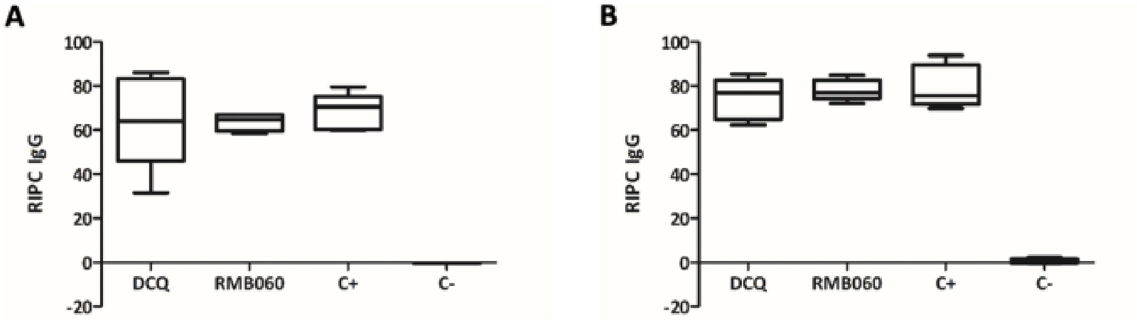

Figure S6: *N. caninum* IgG antibody titers of non-pregnant mice (A) and dams (B) measured from serum collected at the end of the experiment. Results are depicted as the mean of RIPC (relative index per cent) compared to the positive control (C+). In non-pregnant mice and dams, no statistically significant differences in the IgG antibody titers were observed between DCQ- and RMB060-treated mice compared to the C+ group.
